# ALOX15 links lipid metabolism to receptor trafficking in platelet activation

**DOI:** 10.1111/febs.70637

**Published:** 2026-06-29

**Authors:** Yosef Eshetie Amare, Sharad Saxena, Serena Zacchigna

**Affiliations:** ^1^ Cardiovascular Biology Laboratory International Centre for Genetic Engineering and Biotechnology (ICGEB) Trieste Italy; ^2^ Doctoral Study Programme of Molecular Genetics and Biotechnology, Gradute School University of Nova Gorica Nova Gorica Slovenia; ^3^ Department of Medicine, Surgery and Health Sciences University of Trieste Trieste Italy

**Keywords:** ALOX15, lipid metabolism, PAR4, Platelets, receptor trafficking

## Abstract

Platelet activation is traditionally viewed as a signaling process initiated by receptor engagement. In this issue of *The FEBS Journal*, Govatati et al. identify an additional regulatory layer linking lipid metabolism to receptor trafficking. The authors show that ALOX15‐derived lipid mediators promote PKCθ‐NMT1‐dependent myristoylation of PAR4, thereby facilitating its membrane localization and platelet activation. These findings position receptor lipidation and trafficking as active regulatory mechanisms in platelet activation and suggest new opportunities for therapeutic modulation of hemostasis.

## Graphical abstract

This schematic summarizes known and newly identified mechanisms of thrombin‐mediated platelet activation. In addition to classical thrombin‐induced PAR4 signaling pathways, thrombin activates an ALOX15–HETE–PKCθ–NMT1 signaling cascade that promotes protein myristoylation, facilitates receptor trafficking, and enhances PAR4 surface expression on platelets. These findings identify a previously unrecognized upstream regulatory mechanism linking thrombin signaling to receptor availability and platelet activation, expanding current understanding of thrombin‐mediated platelet regulation.

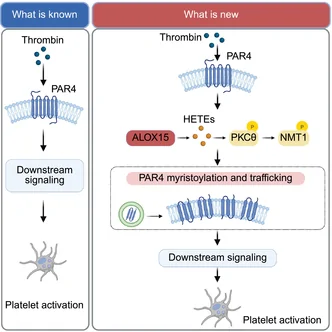

**Abbreviations**
ALOX15: 12/15‐lipoxygenase
GPCR: G protein‐coupled receptor
HETE: hydroxyeicosatetraenoic acid
NMT1: N‐myristoyltransferase 1
PAR: protease‐activated receptor
PKCθ: protein kinase C theta

## Introduction

### Hemostasis and platelet function in disease

Hemostasis is a regulated physiological process that maintains vascular integrity by preventing bleeding while limiting excessive thrombosis. Platelets play a central role in this process by adhering, activating, and aggregating at sites of vascular injury. Dysregulated platelet function contributes to a range of clinical conditions, including bleeding disorders and arterial thrombosis [1, 2].

Platelet activation is a coordinated process involving receptor engagement, intracellular signaling, and cytoskeletal reorganization. Following vascular injury, platelets respond to exposed subendothelial components and soluble agonists, such as thrombin, adenosine diphosphate (ADP), and thromboxane A_2_. These signals converge to promote platelet aggregation and thrombus formation [1, 3]. Although the molecular basis of these processes has been extensively investigated, most models have focused on signaling pathways downstream of receptor activation, with less attention given to the regulation of receptor localization at the plasma membrane [4].

This distinction is relevant because receptor availability at the cell surface can influence both the magnitude and duration of platelet responses. In other cellular systems, receptor trafficking and subcellular localization are recognized as key determinants of signaling dynamics [5]. However, their role in platelet biology, particularly in relation to lipid‐dependent regulatory pathways, remains poorly defined.

### Current understanding and clinical limitations

Platelet activation is mediated by surface receptors, including integrins and G protein‐coupled receptors (GPCRs). Among GPCRs, protease‐activated receptors (PARs) are major mediators of thrombin signaling. PAR1 and PAR4 in humans, and PAR3/PAR4 in mice, function as thrombin receptors and contribute to platelet activation [6, 7]. These pathways regulate intracellular calcium mobilization, granule content secretion, and integrin activation, ultimately leading to thrombus formation [3].

Despite detailed characterization of platelet signaling mechanisms, the clinical management of thrombotic disorders remains challenging. Aspirin inhibits cyclooxygenase‐1 (COX‐1), thereby suppressing thromboxane A_2_ production, whereas P2Y_12_ receptor inhibitors block ADP‐mediated signaling and subsequent integrin activation. Although effective in reducing thrombotic events, both classes of agents are associated with an increased risk of bleeding and do not fully account for interindividual variability in patient response [8]. Moreover, the PAR1 antagonist vorapaxar, which targets thrombin‐mediated platelet activation, is associated with an increased risk of intracranial bleeding. These observations indicate that pathway selectivity does not necessarily translate into a broader therapeutic window [8], in part because platelet activation involves complex and overlapping signaling networks [3, 4]. Consequently, increasing attention has been given to regulatory mechanisms that act upstream of classical signaling pathways, including processes that control receptor availability at the plasma membrane. Regulation of receptor localization may therefore influence platelet responsiveness without directly inhibiting downstream signaling events.

### Lipid metabolism and receptor trafficking: Mechanistic insight

In this issue of *The FEBS Journal*, Govatati *et al*. describe a mechanism linking lipid metabolism to receptor trafficking in platelets [9]. The study identifies 12/15‐lipoxygenase (ALOX15) as a regulator of platelet function and proposes that lipid‐derived mediators influence receptor localization [Fig. 1].

**Fig. 1:**
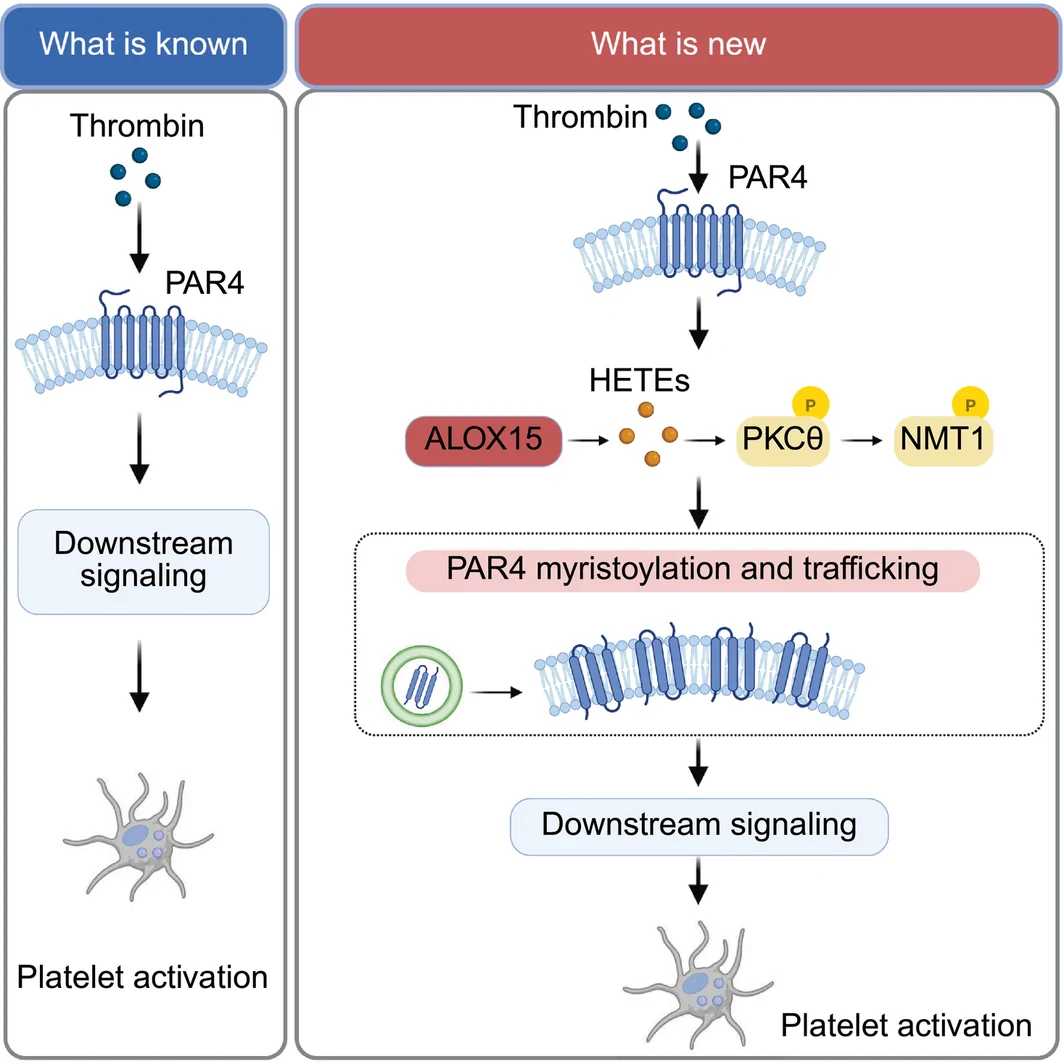
Schematic illustration of known and newly identified mechanisms of thrombin‐mediated platelet activation. In addition to classical thrombin‐induced PAR4 downstream signaling pathways, thrombin activates an upstream ALOX15–HETE–PKCθ–NMT1 signaling cascade that promotes PAR4 myristoylation, facilitates its trafficking, and enhances PAR4 membrane localization on platelets.

Lipoxygenases are enzymes that metabolize polyunsaturated fatty acids to generate bioactive lipid mediators, including hydroxyeicosatetraenoic acids (HETEs) [10]. These molecules have been implicated in platelet activation, primarily by modulating signaling pathways [11]. The study by Govatati *et al*. extends this concept by showing that lipid mediators also regulate receptor trafficking [9].

The proposed mechanism involves a signaling cascade in which thrombin‐induced ALOX15 activity generates HETEs that activate PKCθ, leading to NMT1 phosphorylation and PAR4 myristoylation, a post‐translational modification known to influence membrane localization [12, 13]. This lipid modification promotes PAR4 membrane localization and facilitates PAR3/PAR4 (mouse) or PAR1/PAR4 (human) receptor interactions, thereby enhancing thrombin signaling and platelet activation [9]. Disruption of components of this pathway, including ALOX15, PKCθ, or NMT1, reduces PAR4 trafficking and attenuates platelet activation in the experimental systems examined. These findings establish a mechanistic link between lipid metabolism, post‐translational modification, and receptor localization.

### Implications and perspectives

The study reported by Govatati *et al*. suggests that receptor availability at the plasma membrane represents an important determinant of platelet activation [9]. In addition to ligand binding and downstream signaling, platelet responsiveness may depend on mechanisms that regulate receptor trafficking and membrane localization. Within this framework, lipid metabolism emerges as a potential regulator of receptor positioning and signaling competence.

This concept expands current models of platelet activation, which have primarily focused on signaling events downstream of receptor engagement. The study introduces receptor lipidation and trafficking as additional regulatory layers controlling receptor availability. Similar mechanisms have been described in other GPCR systems, in which receptor trafficking and subcellular localization influence signaling dynamics [5, 14], although their relevance to platelet biology has not been fully explored.

Further studies will be required to define the physiological relevance and broader applicability of this mechanism. It remains unclear whether lipid‐dependent myristoylation extends to other platelet receptors or is specific to PAR4. While the study links PAR4 trafficking to enhanced platelet activation, its impact on established signaling pathways, including calcium mobilization, granule secretion, and integrin activation, remains to be fully characterized. The extent to which this mechanism contributes to platelet regulation under pathological conditions, such as cardiovascular disease or metabolic disorders, also warrants investigation.

From a therapeutic perspective, targeting pathways that regulate receptor trafficking may offer an alternative strategy for modulating platelet function. Unlike current therapies that directly inhibit downstream signaling pathways, modulation of receptor availability could alter platelet responsiveness through a distinct mechanism. Whether such approaches can achieve effective antithrombotic activity while preserving hemostatic balance remains to be determined.

In summary, the work discussed here identifies a mechanistic link between lipid metabolism and receptor trafficking in platelet activation. By showing that lipid‐derived mediators influence receptor post‐translational modification and membrane localization, the study advances our understanding of platelet regulation. These findings suggest that control of receptor availability may represent an important regulatory mechanism in platelet biology, with broader implications for GPCR‐mediated signaling processes.

## Conflicts of interest

The authors declare no conflicts of interest.

## Author contributions

YA wrote the manuscript and prepared the figure. SS prepared the figure. SZ supervised, reviewed, and edited the manuscript. All authors have read and approved the final manuscript for publication.
